# P-2072. Sociodemographics and Viral Load Outcomes of People Newly Diagnosed with HIV or Returning to HIV Care Receiving RapidTx Cards at NYC Health+Hospitals/Bellevue

**DOI:** 10.1093/ofid/ofaf695.2236

**Published:** 2026-01-11

**Authors:** Ian Maynor, Abigail Smith, Cooper Urban, Emma R Boockvar, Robert Pitts, Shelly Blumenthal, Kathryn Jano, Shree Sundaresh, Ofole Mgbako

**Affiliations:** NYC Health+Hospitals/Bellevue, New York, NY; NYC Health+Hospitals/Bellevue, New York, NY; New York University Grossman School of Medicine, North Java, NY; New York University Grossman School of Medicine, North Java, NY; NYU Langone Health, New York, New York; NYC Health+Hospitals/Bellevue, New York, NY; New York University Langone Health, New York, New York; Infectious Diseases Clinical Fellow, Yale School of Medicine, New Haven, Connecticut; NYC Health+Hospitals, Brooklyn, NY

## Abstract

**Background:**

Immediate antiretroviral therapy (iART) is ART initiation immediately after HIV diagnosis or upon care linkage. In New York City (NYC), RapidTx cards cover the cost of 30 days of ART for un- or underinsured patients via the New York State (NYS) AIDS Institute HIV Uninsured Care Program. This study characterizes sociodemographics and viral load suppression (VLS) of patients initiated on iART via RapidTx cards at NYC Health+Hospitals/Bellevue.

**Figure:**
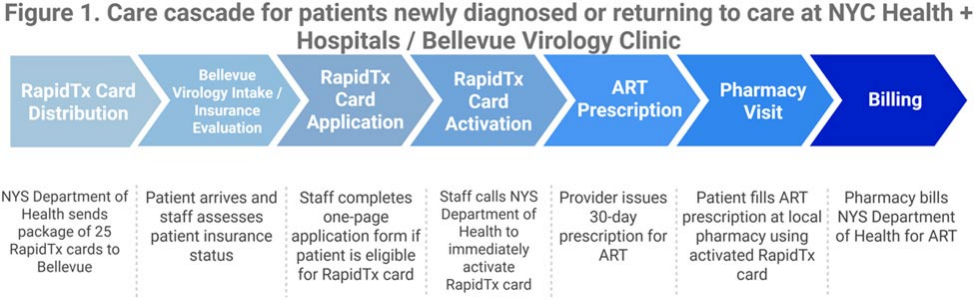

**Figure:**
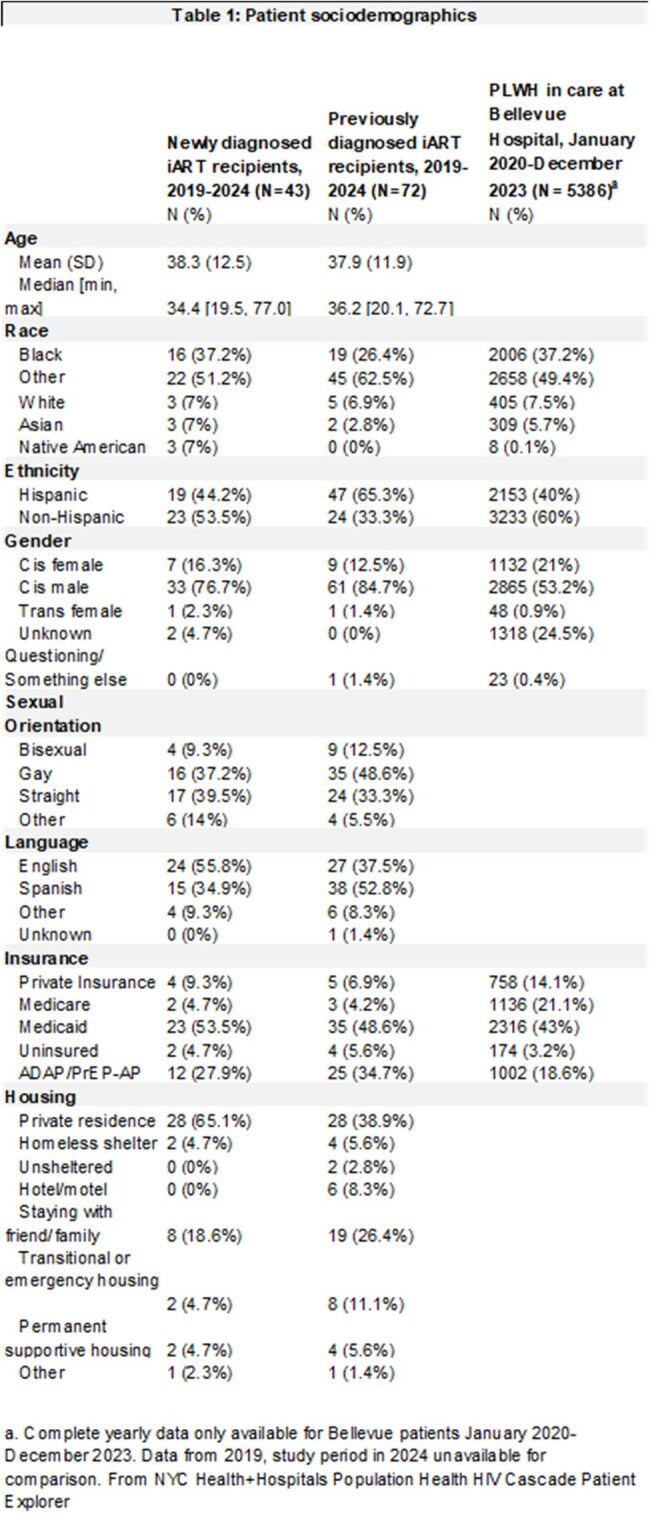

**Methods:**

We conducted a single-center, retrospective study of 115 patients issued RapidTx cards from January 1, 2019 to July 31, 2024. The primary outcome was VLS at 3 months after care linkage at the subsequent Bellevue HIV/virology appointment for patients receiving RapidTx cards. Descriptive statistics were used for socio-demographics and clinical characteristics. We compared the Rapid Tx cohort’s sociodemographics to those of all Bellevue patients with HIV from 2020-2023. We then compared the primary outcome to VLS for Bellevue patients newly diagnosed with HIV in 2023.

**Figure:**
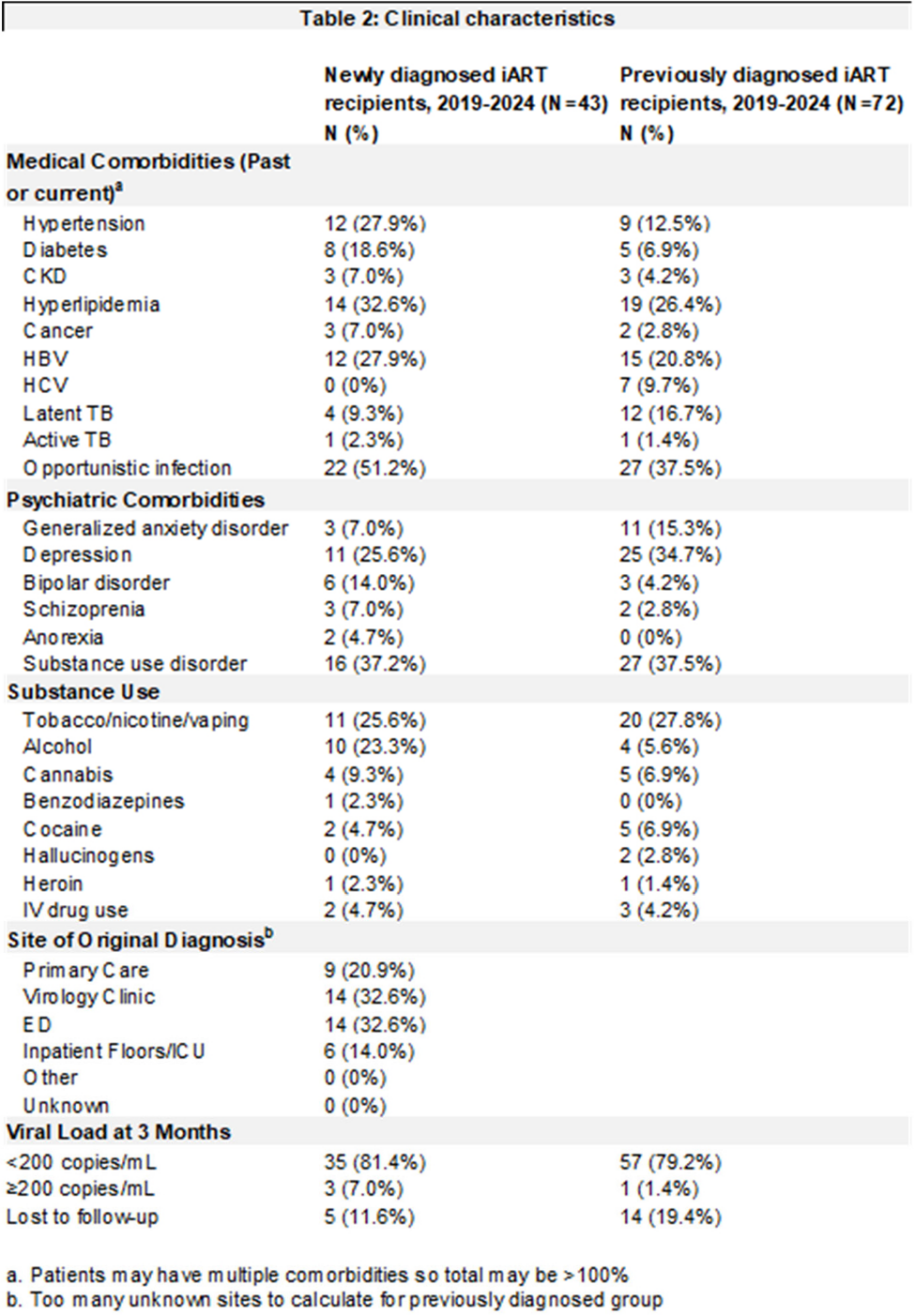

**Results:**

Out of 115 participants, 37.39% (N=43) were newly diagnosed with HIV at Bellevue. 62.61% (N=72) were diagnosed in the past and returning to care. Figure 1 summarizes the RapidTx card workflow. Table 1 summarizes sociodemographics and Table 2 summarizes clinical characteristics. RapidTx patients were more often Hispanic, cisgender male, uninsured or on Medicaid than the general Bellevue population with HIV. 83.48% (N=96) had viral load testing 3 months after care linkage; 14.78% (N=17) were lost to follow-up. 81.39% (N=35) of newly diagnosed patients and 79.16% (N=57) of patients returning to care had VLS at 3 months. For patients with new HIV diagnoses in 2023, 54% had VLS at 3 months at Bellevue while 51% had VLS at 3 months in NYC.

**Conclusion:**

RapidTx cards were an effective tool for iART initiation with better initial VLS outcomes for RapidTx patients than for newly diagnosed patients at Bellevue and in NYC. There was no difference in VLS between new and previously diagnosed RapidTx patients. Future studies should prioritize more support for RapidTx patients prior to them becoming lost to follow-up.

**Disclosures:**

Robert Pitts, MD MPH, Gilead Inc: Advisor/Consultant|ViiV: Advisor/Consultant Ofole Mgbako, MD, MS, Gilead Sciences: Advisor/Consultant

